# *In Silico* Generation of Alternative Hypotheses Using Causal Mapping (CMAP)

**DOI:** 10.1371/journal.pone.0005378

**Published:** 2009-04-29

**Authors:** Gabriel E. Weinreb, Maryna T. Kapustina, Ken Jacobson, Timothy C. Elston

**Affiliations:** 1 Department of Cell and Developmental Biology, University of North Carolina at Chapel Hill, Chapel Hill, North Carolina, United States of America; 2 Department of Pharmacology, University of North Carolina at Chapel Hill, Chapel Hill, North Carolina, United States of America; 3 Lineberger Cancer Center, University of North Carolina at Chapel Hill, Chapel Hill, North Carolina, United States of America; Center for Genomic Regulation, Spain

## Abstract

Previously, we introduced causal mapping (CMAP) as an easy to use systems biology tool for studying the behavior of biological processes that occur at the cellular and molecular level. CMAP is a coarse-grained graphical modeling approach in which the system of interest is modeled as an interaction map between functional elements of the system, in a manner similar to portrayals of signaling pathways commonly used by molecular cell biologists. CMAP describes details of the interactions while maintaining the simplicity of other qualitative methods (e.g., Boolean networks).

In this paper, we use the CMAP methodology as a tool for generating hypotheses about the mechanisms that regulate molecular and cellular systems. Furthermore, our approach allows competing hypotheses to be ranked according to a fitness index and suggests experimental tests to distinguish competing high fitness hypotheses. To motivate the CMAP as a hypotheses generating tool and demonstrate the methodology, we first apply this protocol to a simple test-case of a three-element signaling module. Our methods are next applied to the more complex phenomenon of cortical oscillations observed in spreading cells. This analysis produces two high fitness hypotheses for the mechanism that underlies this dynamic behavior and suggests experiments to distinguish the hypotheses. The method can be widely applied to other cellular systems to generate and compare alternative hypotheses based on experimentally observed data and using computer simulations.

## Introduction

Recently much effort has been focused on gaining a systems-level understanding of processes that occur on the cellular and molecular level. Because the external and internal environments of cells are constantly changing, any design principle employed at this level must be robust to perturbations. In terms of computational models, this implies that some degree of uncertainty in key parameter values must be tolerated without significantly affecting system performance. This situation leads quite naturally to an increased role of coarse-grained descriptions of cellular systems such as Boolean networks [1]–[5] or Dynamic Bayesian Networks [6]–[14], that do not require the precision of detailed biophysical models.

Previously we proposed a graphical systems biology approach, causal mapping (CMAP), to describe complex cellular and molecular systems [15]. CMAP is a course-grained biological network tool that takes into account causal interactions between network elements and provides a description of the overall system dynamics. The network of interest is modeled as a map based on known and hypothetical interactions between elements of the system, in a manner similar to common portrayals of signaling pathways. CMAP provides an intuitive algorithm for evolving the values of the elements in time based on the interactions between the elements. The CMAP maintains the simplicity of other course-grained methods, including Boolean networks, but there are essential differences. The elements of the CMAP, which are referred to as concepts, vary continuously in time between the values of 0 and 1. The strength of the interactions between elements, called weights, ranges from [−1, 1]. By contrast, for Boolean networks, the values of the nodes, which are analogous to concepts in the CMAP methodology, vary discretely between 0 and 1 and the strength of the interactions are restricted to 0, 1 or −1. In classical Boolean networks (see, for example, [16]) a node does not change its value unless the inputs to that node exceed a threshold. By contrast, CMAP concepts evolve in time as long as they are acted upon by other concepts. In CMAP, the strength of the interactions between concepts is determined by a set of weights (Appendix A)that can be interpreted in linguistic terms such as ‘strong’, ‘weak’, and ‘moderate’ [15]. This approach simplifies the CMAP analysis (see below) by limiting the parameter space of the models.

In this paper, we introduce the use of the CMAP as a hypothesis generating tool. Other similar approaches have been developed using different network techniques including Boolean networks [1]–[5]. First, we use a simple example of a three-node network to demonstrate how the CMAP can be used to generate hypotheses for pathway architectures that generate transient responses [17]–[23]. This type of behavior occurs in signal pathways that become desensitized or adapt to persistent stimuli. Then we apply hypotheses generation to the problem of cortical oscillations of spreading cells [15], [24]. Our goal is to develop a tool to investigate the behavior of living systems and to provide substantial guidance to experimentalists. We show how CMAP can be employed: (i) to develop several hypotheses that satisfy criteria which are based on experimental observations; (ii) to rank those hypotheses in terms of how well they satisfy the criteria; and (iii) to make testable predictions that distinguish between the highest ranking hypotheses.

## Results

### Illustration of the method: a three-concept system

To illustrate the algorithm, we considered a simple CMAP consisting of three concepts (C_1_–C_3_) with the goal of determining which network architectures are capable of adaptation. That is, at least one of the concepts must return to near its basal level in the presence of a persistent stimulus. Such behavior is common in genetic networks and signaling pathways [17]–[23]. We defined criteria for successful configurations in the following way: using an initial value of concept 1 (C_1_) of 1 concept 3 (C_3_) was required to respond transiently and reach a maximum value higher than 0.2, and eventually return to a value below half of the maximum. Note that the fitness indices below correspond to calculations that satisfy these particular criteria; the optimum scheme in terms of highest fitness will, in general, depend on the selection of criteria.

**Figure 1:**
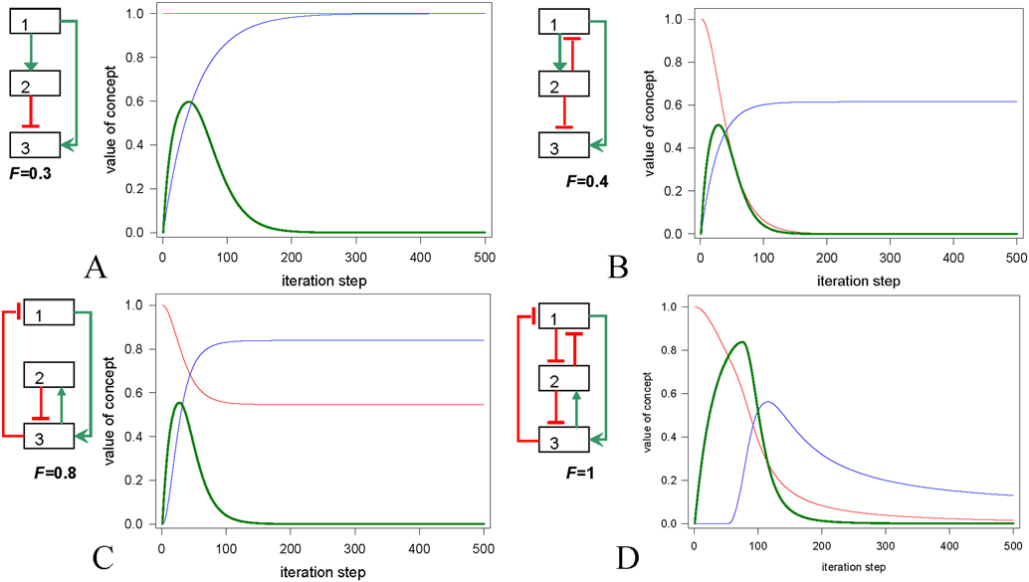
Simple three-element CMAP configurations. Depictions of the configurations, with the concepts in boxes and their influences represented by green arrows (positive) and bar-headed red connectors (negative), are shown on the left of each panel. All configurations have the requirement that concept 1 transiently activates concept 3. *F* denotes fitness index for corresponding configuration. On the right of each panel, the time-courses of the concept values are shown: red, C_1_; blue, C_2_; and dark green, C_3_. A: only feed-forward reactions are allowed; B: a feedback from C_2_ to C_1_ is allowed; C: a case where no interactions between C_1_ and C_2_ are allowed; D: there are no limitations on the connections. (See Illustration of the Method.)

In Fig. 1 a sequence of CMAPs in order of increasing fitness index is presented. The fitness index corresponds to the fraction of parameter space that generates simulation results consistent with the criteria for an acceptable configuration (See Methods for the formal definition). Initially, we assumed a feed-forward architecture, so that configurations where C_2_ influenced C_1_ or/and C_3_ influenced C_1_ or C_2_ were not considered (Fig. 1A). Each of the three allowed influences can be positive, negative or zero. The strength of each influence is characterized by a weight. Each weight represents a free model parameter. The weights are restricted to a finite range of discrete values. The number of these values is denoted by the set size K. For K = 5, the range of values for the weights is ±[0.1, 0.3, 0.5, 0.7, 0.9]. There are 125 combinations of parameter values for configurations restricted to only a single influence between any two concepts and therefore for three weights: K^3^ = 125. The only feed-forward configuration satisfying the criteria is shown in Fig. 1A. This architecture represents an incoherent feed-forward loop and is well-known in the systems biology literature [25]. Our simulations showed that only about 30% of the parameter combinations produced the required behavior giving a fitness index of 0.3. Typical time series for the concepts are shown in the right panel of Fig. 1A.

When a negative feedback loop from C_2_ to C_1_ (Fig. 1B) is included in the system, the resulting configuration had a larger fitness index (*F* = 0.4). In this configuration the behavior of concept 1 is qualitatively different from the previous one (compare Fig 1A and 1B) enabling the two hypotheses to be distinguished experimentally by measuring C_1_. If there are no interactions between C_1_ and C_2_, the configuration depicted in Fig. 1C has the highest fitness (0.8). In this case, C_1_ diminishes with time to a steady-state level. If there are no restrictions on any of the connections, a configuration (Fig. 1D) can be found which satisfies the criteria for any combination of the weights (*F* = 1). In this case, because of the competition between C_1_ (inhibiting) and C_3_ (activating), C_2_ evolves non-monotonically in time. Since none of the other hypotheses produced similar results, observation of this behavior would provide strong support for such a mechanism. This behavior is a general property of the model but, for some parameter sets, it is not so pronounced and its detection would require adequate signal to noise ratio in the experiment. In summary, in this section we showed how the hypotheses generation algorithm can be applied to a simple three-concept system to determine pathway architectures that respond transiently to a sustained external signal.

### Hypothesis generation for cortical oscillations

Pletjushkina et al. [24] observed that spreading epithelial cells or fibroblasts, in which the microtubules have been depolymerized, undergo rhythmic oscillations of the cell body that last for several hours. The complex nature of this system makes it a good candidate for hypothesis generation using the CMAP. It is known that the oscillations involve intracellular calcium and activation of the Rho pathway, which occurs following microtubule depolymerization [24].

**Figure 2:**
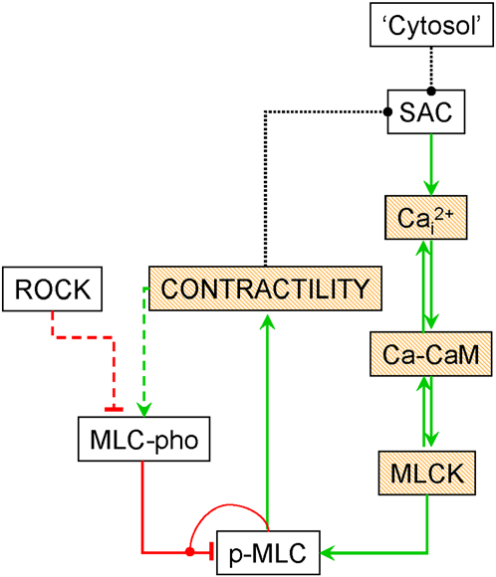
CMAP of cortical oscillations for hypotheses generation. The scheme is derived from the original CMAP paper [15] to describe the phenomenon of cortical oscillations [24]. The red connectors depict the inhibitory influences that are characterized by negative weights while the green arrows represent activation characterized by positive weights. The black dotted connectors reflect the unknown causal influences being tested in this work: they can be activation (green) or inhibition (red). The shadowed boxes indicate ‘self-inhibitions’ for a corresponding concept *C_i_* where *w_ii_*<0. The colored dashed connectors can be characterized by corresponding sign of weight or be non-existent during testing. The red half-circle connected to the inhibitory influence between MLC-pho and p-MLC depicts the second order interaction between the product (p-MLC) and the enzyme (MLC-pho). (For explanations of higher order interactions see Materials and Methods).

In a previous study [15], we used CMAP to propose a mechanism for the generation of cortical oscillations that involved a negative feedback loop in which myosin-based contractility negatively regulated stretch activated calcium channels (SACs). The SACs opened due to stretching of the cell surface when the cytosol moves from one side of the cell to the other [26]. The CMAP model assumes that the role of Rho pathway is to decrease the level of myosin light chain phosphatase (MLC-pho) [15]. This is because active Rho activates Rho kinase (ROCK) which phosphorylates MLCpho, a negative regulator of myosin, thereby inactivating this enzyme and simultaneously increasing the level of phosphorylated myosin light chain and increasing actomyosin contractility. A mechano-chemical model using a system of ordinary differential equations was developed based on that CMAP configuration that recapitulated the experimental results and made testable predictions [26]. Note that in both cases we employed non-spatial models; this simplification applied because of the symmetry of the oscillating cell in which the two opposing sides oscillate out of phase with each other [26]. To take into account the volume conservation and cytosolic movement, we introduced constant force acting on the membrane (‘Cytosol’→SAC in Fig. 2). Independently, another group [27] described a similar oscillatory phenomenon using a mechano-chemical description in which a negative feedback from contractility to stretch activated channels is invoked. However, other mechanisms may be responsible for generating cortical oscillations. In this paper we demonstrate how the CMAP method can be used to evaluate alternative hypotheses.

#### Candidate systems

We constructed eight different candidate configurations of the system, all of which include eight concepts (see Fig. 2 and Table 1), including the one previously tested [15]. In our previous model, it was assumed that the Rho pathway was not influenced by the other elements of the system. Therefore, we decided to relax this assumption and test for a possible feedback loop from contractility to the Rho pathway. For simplicity, rather than modeling the entire Rho pathway, we assumed that contractility directly influences the myosin light chain phosphatase (MLC-pho). This feedback is an alternate to the ‘contractility→SAC’ feedback. The eight candidate configurations varied by the nature of the four influences: “‘Cytosol’→SAC”, “contractility→SAC” (depicted as black connectors in Fig. 2), “contractility→MLC-pho”, and “ROCK→MLC-pho” (color coded connectors, see legends to Fig. 2). The sign of these influences for each configuration are presented in Table 1 in the second half of column 2. If an influence is not included in the particular configuration, it is marked as ‘0’. The rest of the influences are considered to be known and are held fixed in the initial investigations.

**Table 1:** Results of hypothesis generation^1^.

Configuration #	Hypothesis description		Total number of combinations, *P_total_*		Number of valid combinations, *P_i_*		Fitness index, F		Set size, K
			Fixed	Random	Fixed	Random	Fixed	Random	
1	2		3	4	5	6	7	8	9
1	Con to SAC	**−**	19683	4000000	0	6	0	1.50E-06	3
	‘Cytosol’ to SAC	**+**	1953125	4000000	292	97	1.50E-04	2.43E-05	5
	Con to MLC-pho	**−**	40353607	4000000	4548	222	1.13E-04	5.55E-05	7
	ROCK to MLC-pho	**+**							
2	Con to SAC	**+**	19683	4000000	0	0	0	0	3
	‘Cytosol’ to SAC	**−**	1953125	4000000	0	0	0	0	5
	Con to MLC-pho	**−**	40353607	4000000	0	0	0	0	7
	ROCK to MLC-pho	**+**							
3	Con to SAC	**+**	19683	4000000	0	0	0	0	3
	‘Cytosol’ to SAC	**−**	1953125	4000000	0	0	0	0	5
	Con to MLC-pho	**+**	40353607	4000000	0	0	0	0	7
	ROCK to MLC-pho	**−**							
4	Con to SAC	**−**	19683	4000000	279	7916	0.0142	1.98E-03	3
	‘Cytosol’ to SAC	**+**	1953125	4000000	3429	4250	1.76E-03	1.06E-03	5
	Con to MLC-pho	**+**	40353607	4000000	91854	2945	2.28E-03	7.36E-04	7
	ROCK to MLC-pho	**−**							
5	Con to SAC	**−**	2187	4000000	743	185617	0.3397	0.0464	3
	‘Cytosol’ to SAC	**+**	78125	4000000	8127	127856	0.104	0.032	5
	Con to MLC-pho	**0**	823543	4000000	71221	43949	0.0865	0.011	7
	ROCK to MLC-pho	**0**							
6	Con to SAC	**+**	2187	4000000	0	0	0	0	3
	‘Cytosol’ to SAC	**−**	78125	4000000	0	0	0	0	5
	Con to MLC-pho	**0**	823543	4000000	0	0	0	0	7
	ROCK to MLC-pho	**0**							
7	Con to SAC	**0**	6561	4000000	0	0	0	0	3
	‘Cytosol’ to SAC	**0**	390625	4000000	0	0	0	0	5
	Con to MLC-pho	**+**	5764801	4000000	0	0	0	0	7
	ROCK to MLC-pho	**−**							
8	Con to SAC	**0**	2187	4000000	0	0	0	0	3
	‘Cytosol’ to SAC	**0**	78125	4000000	0	0	0	0	5
	Con to MLC-pho	**−**	823543	4000000	0	0	0	0	7
	ROCK to MLC-pho	**+**							

1 The weight for the following influences were fixed: MLCK-p-MLC, MLC-pho - p-MLC, calcium uptake and Ca-pump work (calcium - calcium), calcium release from Ca_i_^2+^-CaM (Ca-CaM – Ca _i_^2+^), Ca-CaM – MLCK, Ca-CaM dissociation (Ca-CaM - Ca-CaM), MLCK-Ca-CaM dissociation (MLCK - Ca-CaM).

#### Criteria for evaluating hypotheses

The configurations were tested against three criteria formulated from experimental observations [24]. First, after depolymerization of microtubules, spreading cells exhibited morphological oscillations. Second, the morphological oscillations were accompanied by oscillation of intracellular calcium. Finally, oscillatory behavior was halted by inhibition of Rho kinase (ROCK).

#### Testing the configurations

The configurations were tested in a way similar to that described in the section Illustration of the Method. Configurations 1–4, 7, and 8 have a total of sixteen influences, configurations 5 and 6 have fourteen. First, we fixed the seven weights relating to the calcium pathway (see footnote for Table 1). The values for these weights were taken from our previous work [15]. We simulated all configurations with all possible sets of weights for the influences that were not fixed and tested the results against the experimentally determined criteria. In addition, each set of weights was simulated with different set sizes (K = 3, 5, 7). Column 7 in Table 1 shows the fitness indices obtained for each configuration when these seven weights were fixed. Configurations 4 and 5 have the highest fitness indices and qualify as hypotheses (Table 1). It is noteworthy that both hypotheses suggest that the oscillatory behavior observed by Pletjushkina et al [24] requires a negative feedback from contractility to stretch activated calcium channels (SAC).

#### Monte-Carlo method for sampling parameter space

In the previous section we discussed the results of simulations with fixed values for several weights. based on the results of our previous work [15]. To test the validity of fixing these weights, we performed simulations in which the values of all the weights are allowed to vary. To do this, we used a Monte Carlo approach to generate 4,000,000 candidate sets out of the >10^11^ possible combinations of parameter values. The value of each weight was chosen from a uniform random distribution. The results from this approach are presented in Table 1 (columns 4, 6, and 8) and confirm the analysis presented in the previous section.

**Figure 3:**
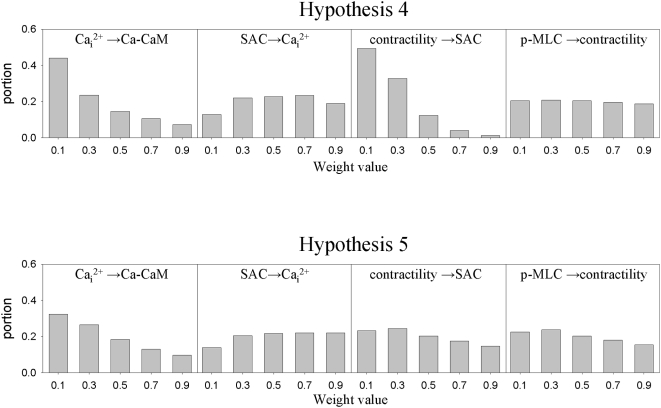
Distribution of weights for selected causal influences for different hypotheses. Distribution of weights for hypotheses 4 and 5 for four causal influences. Top row: hypothesis 4; bottom row: hypothesis 5. All histograms are normalized by the total number of occurrences.

We constructed histograms of the distribution of weights associated with each causal influence that give successful outcomes for hypotheses 4 and 5 based on the Monte-Carlo method for sampling parameter space. The results for four selected causal influences are shown in Fig. 3. Note that all values of the weights produced positive outcomes, which implies that the parameter search could not be limited to a subset of the parameter space. Another feature of the histograms is that some weights have fairly uniform distributions (p-MLC→contractility for hypothesis 4) while others are more localized around a specific value (contractility - SAC in hypothesis 4). Those distributions that exhibit ‘flatness’ reflect robustness of the system with respect to the interaction in question. That is, no matter how strong or weak this interaction is there are always parameter values that lead to oscillations.

**Figure 4:**
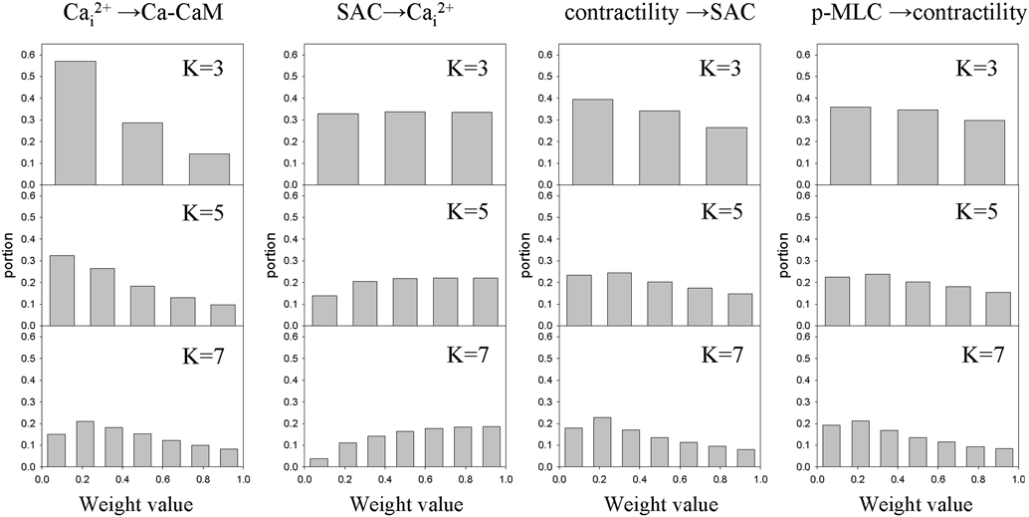
Distribution of weights for selected causal influences for different set sizes. Distribution of weights for hypothesis 5 for four causal influences as a function of set size. K = 3 (upper row), 5 (middle row), 7 (bottom row). All histograms are normalized by the total number of occurrences.

We tested different values of the set size (K) to verify that our conclusions were independent of this parameter. Figure 4 shows histograms based on Monte-Carlo simulations for K = 3, 5, and 7. The histograms show similar shapes of weight distributions for different K values for four causal influences: contractility→SAC; SAC→Ca_i_^2+^; calcium→calmodulin; and p-MLC→contractility (see Fig. 3)). Table 1, column 8, confirms that the fitness indices lead to the same hierarchy of configurations for all three K values: hypothesis 5>hypothesis 4>hypothesis 1. These comparisons of the results for different K values suggested that the results of simulations should not be greatly influenced by the choice of the set size.

#### *In silico* experiments to refine predictions

A key problem in modeling is to find parameter sets that describe system behaviour. We used the CMAP approach to find parameter sub-space whose values describe qualitative features of experimental data sets. This led to computation of the fitness index for various configurations (see Table 1) from which hypotheses were defined as those configurations with *F*>0. For hypothesis 5 (K = 5), for example, we got 127,856 valid weight combinations out of 4,000,000 we tried. This result raises several questions. How many out these combinations really actually describe the oscillating cells and how can hypotheses be differentiated?

To further test competing hypotheses, we asked how weight sets included in valid hypotheses would respond to defined perturbations that correspond to feasible manipulations of the experimental system under investigation. To quantify the effects of such perturbations, we adopted the following procedure:

- Identify an influence which can be experimentally manipulated, e.g. titration of an inhibitor;
- For each set of weights that produced oscillations, shift the weight of interest by a chosen amount to simulate the experimental manipulation;
- Simulate the CMAP with the new set of parameters and determine the period and amplitude of the resulting oscillations and the fraction of non-oscillators;
- Compute the number of sets of weights in the ensemble that lead to an increase, decrease, or a loss of oscillations.

**Figure 5:**
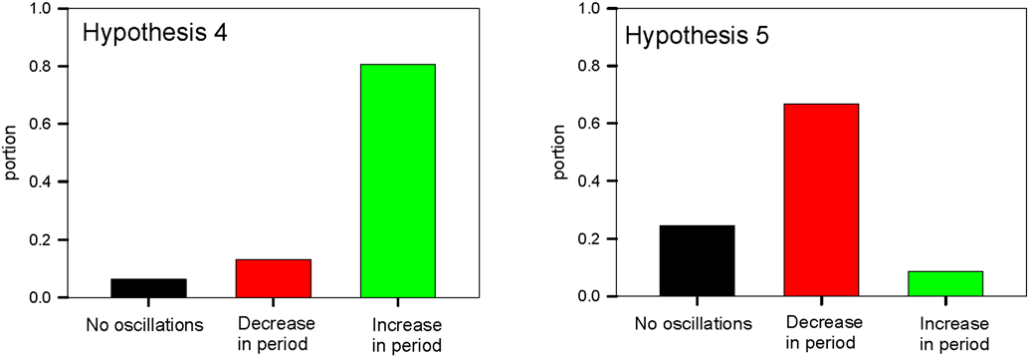
Results of *in silico* experiments to test the effect of reducing cytoplasmic free calcium according to Hypotheses 4 and 5 (see text). The bar graphs represent the portion of weight sets that produced an increase in oscillation period (green) or a decrease in oscillation period (red) or cessation of oscillations (black) when the value of calcium self-inhibition weight (*w_CaCa_*<0) was decreased by 0.4 from the initial value for each set with the constraint that the final weight value could not be less than −0.9. Weight sets that already had the minimum value of −0.9 (maximum ‘self-inhibition’) were excluded from simulations. All initial weight sets were taken from the Monte Carlo simulations.

This procedure is demonstrated in Figure 5 for the case where the calcium self-inhibition [15] is perturbed. The cytoplasmic free calcium is determined by a number of factors including calcium influx, efflux and the status of internal endoplasmic stores. Thus, for example, cytoplasmic free calcium can be experimentally reduced by introducing calcium buffers into the cell. To reproduce this experimental manipulation in the CMAP calculation, the calcium self-inhibition was strengthened by shifting the corresponding weight by −0.4 in all successful sets previously produced by Monte Carlo simulation. After recalculation with new parameters, Hypothesis 4 predicted that the population average period will rather increase when cytoplasmic free calcium is reduced; by contrast, Hypothesis 5 suggested the opposite (Figure 5).

## Discussion

The causal mapping technique has the potential to be an effective tool for studying complex biological systems. On the one hand, CMAP is a semi-quantitative method similar to Boolean networks and its extensions [16]. On the other hand, CMAP provides a more detailed description than other graphical approaches with similarities to the difference equation approach. Thus, in terms of modeling techniques, the CMAP technology occupies an intermediate position between purely graphical methods and more quantitative models based on either ordinary or partial differential equations or stochastic formulations and it puts some limitations on possible mechanisms. For example, both mechano-chemical models of cortical oscillations that have been developed recently ([26], [27]) include a negative feedback from contractility to a mechano-sensitive source of calcium such as stretch activated calcium channels (SAC). This feature was predicted by CMAP modeling [1] and suggests that application of the coarse-grained CMAP technology can illuminate key qualitative requirements of mechanisms put forward to account for system behavior.

In this paper, we have added hypotheses generation to the CMAP toolbox. This methodology enables investigators to rank hypotheses according to a fitness index. Hypotheses with high fitness indices represent operating mechanisms that are robust to variations in parameter values, and, therefore, in theory represent good design principles for operating in the fluctuating environments found at the cellular and molecular levels. Thus, one interpretation of a high fitness index is that these systems represent architectures most likely to survive natural selection.

We applied the hypothesis generation tool to a simple test case of a three-element signaling module and to the more complex phenomenon of cortical oscillations [24]. For the former case, we demonstrated that the CMAP protocol can be used to generate pathway architectures capable of adaptation to persistent signal. Intriguingly, our analysis found a configuration that produced adaptation for all parameter values (*F* = 1). It would be interesting to determine if this pathway architecture exists in real signaling or regulatory systems. For the case of cortical oscillations, the two main conclusions are that i) a negative feedback from cell contractility to mechanochemically-activated calcium release is required to qualitatively reproduce experimental observations for this system [26], [27] and ii) that there are possible connections between the Rho pathway and contractility [28], [29] that should be explored experimentally and in future modeling. Our methodology also provides a mechanism for generating experimentally testable predictions to discriminate competing high-fitness hypotheses. An important feature of our approach is that the predictions are not based on perturbations to a single parameter set, but represent trends in the behavior of the hypotheses when all the parameter sets that generate results consistent with experimental data are considered. Because we are able to exhaustively sample the parameter space, a consistency between new experimental results and model predictions is more likely to be indicative of the design architecture of the biological system rather than reflect a particular choice parameter values. While a single experiment may not definitively prove a mechanism, it would reduce the regions of parameter space for various hypotheses that produce behavior consistent with all the experimental results. It may then be possible to find experimental perturbations for which valid hypotheses produce qualitatively different behavior for all parameter values within this restricted space.

Coarse-grained approaches such as this will have some limitations. Of course, as the complexity of biological networks increases, the number of possible configurations increases in an exponential fashion. However, this is limited in a practical sense by the prior knowledge we have about this system derived from laboratory experiments and the biological literature. It could also be argued that the weight interval we employ [−1, 1] is unduly restrictive in limiting the range of variation of weights that we employ. In this regard, it should be noted this is already an improvement in terms of modeling dynamics when compared to the frequently employed Boolean networks which are binary in nature. Moreover, this range of weights employed already produces a rich repertoire of parameter combinations that qualitatively reproduce the observed behavior.

As biologists continue to move toward studying cellular and molecular systems as a whole, there will be an increased need for mathematical approaches to interpret and codify experimental results. We believe the CMAP provides the appropriate level of description within an intuitive framework to make sense of these complex biological systems.

## Materials and Methods

### Description of the method

#### CMAP basics

The equations describing how the concepts *C_j_(t)* evolve in time are [15]:
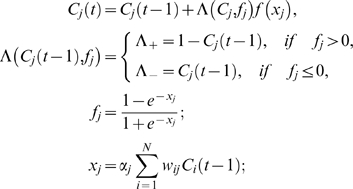
(1)where *N* denotes the number of elements in the system and the *w_ij_*'s are the weights of interactions. The first equation consists of two terms: the value of the concept at the previous time step *C_j_(t−1)* and the product of the causal function *f(x,)*, which determines how the concepts influence *C_j_(t−1)* , and the scaling factor Λ which forces *C_j_(t−1)* to stay within the range between 0 and 1. The coefficient *α_j_* is described below.

#### Weights and the set size

Each causal influence is assigned a weight (*w_ij_*) during the simulations. The set size, K, is the number of intervals used to discretize these weights. As mentioned above, each weight can be positive or negative (a value of zero reflects a non-existent interaction between corresponding concepts). The absolute value of a weight can take a number in the interval [0, 1] determined by the set size K. In our original work, we divided this interval evenly into K subintervals and assigned weight values as the midpoint of the subintervals [15]. For example, if K = 5 then the possible values of the weights *w_ij_* are: ±[0.1, 0.3, 0.5, 0.7, 0.9].

#### Parameter α

In [15] we introduced the parameter *α_i_*, which determines how much the causal function *f(x)* in Eq.(1) can change during single iteration. At maximum possible input, i.e. when all input concepts *C_i_* and the corresponding weights *w_ij_* are equal 1, the value of the causal function is
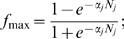
(1)


In the previous work [15] we assumed that this maximum step can not exceed the interval size *p_j_* determined by the set size, i.e. *f_max_* = *p* = 1/K. Thus, from Eq.(1a) we have
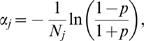
(2)where *Nj* is number of influences on the concept *Cj*. In general, this condition can be relaxed and the role of *α_i_* should be investigated further.

#### Higher order interactions

In the original version of the CMAP [15] the concepts in the exponents of *f(x)* occurred only linear combinations. However, it is clear that higher order terms may also be required. For example, a second order reaction requires that both reactants are present for an interaction to occur. Therefore, we introduce higher order inputs as a generalization of the previous version:
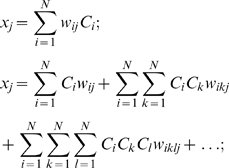
(3)


Using this extension, the causal function for phosphorylated myosin light chain (MLC-pho→pMLC influence, see Fig. 2) has the form
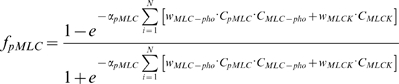
(4)


Note the differences between MLCK→pMLC and MLC-pho→pMLC influences: in the first case pMLC is a product of phosphorylation while in the second, it is a substrate of a dephosphorylation. Assuming a large pool of non-phosphorylated MLC, a substrate for the first reaction, in the cell (meaning no big change in its concentration), there is no need for a second order reaction for MLCK→pMLC influence in contrast to case of MLC-pho→pMLC.

### Definitions

#### CMAP configurations

A given network configuration is defined by the number of concepts (nodes) and influences (edges) between the concepts and the nature of the influences (positive or negative). For a given network configuration, the weights of the influences and the initial values for the concepts can vary, but the weights cannot change sign. Network configurations can differ from each other by the number of concepts, the connectivity or the nature of the influences (positive or negative). For example, Fig. 1 shows 4 different CMAP configurations (A–D), each containing 3 concepts. The configurations differ either by the connectivity of the network or the nature of the influences.

#### Fitness index

Because we assume a discrete range of values for the weights, for any given CMAP configuration, there is a finite number of parameter sets that need to be investigated. This number, *P_total_*, defines the total volume of parameter space for a given configuration. To be a viable hypothesis, a CMAP configuration must reproduce known experimental results for the biological system under investigation. For each candidate parameter set, the output of the CMAP configuration is checked for consistency with experimental results, and if consistent results are obtained, the parameter set is accepted. The fitness index, *F_i_*, is defined as
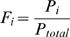
(5)where *P_i_* is the number of parameter sets that are consistent with the experimental behavior. Note that in this work the fitness index is a relative value and should be compared to other indices computed in the same way. Since the total parameter space in our cortical oscillation model is too big for exhaustive simulations (for example, for hypothesis 5 and K = 5, *P_total_* = 5^14^ = 6,103,515,625), we randomly picked 4*10^6^ weight combinations, see column 4 in Table 1.

#### Hypothesis

A CMAP *configuration* is defined as a *hypothesis* only if it has a non-zero fitness index. The larger the fitness index, the larger is the fraction of parameter space for which the configuration meets the experimental criteria. Therefore, the fitness index provides a mechanism for ranking the hypotheses under consideration.

### Simulations

Simulations were performed using Fortran95 to calculate the fitness indices and MATLAB (The Mathworks, Inc) for the rest of simulations.

#### Three-node signaling module

For three-node simulation, we examined all possible CMAP configurations with a full set of weight values. The configuration had a total of six possible influences between concepts for which the weights could be positive, negative or zero. For K = 5, each positive or negative weight could have five different absolute values [0.1, 03, 0.5, 0.7, 0.9]. Each simulation was performed for 5000 “time” steps. The initial parameters for concept values were: C_1_ = 0.5; C_2_ = C_3_ = 0. We considered that a particular set of weights met a criterion when during the simulation the value of C_3_ transiently increased higher than 0.2 with a subsequent decrease to lower than half of the maximum value which was reached during the increasing stage.

#### Cortical Oscillations

During the simulation, when some of the influences were fixed (see Table 1), the remaining influences were used in all possible combinations. In case of Monte Carlo simulations, 4,000,000 weight combinations were checked. In both cases the concept values for 20,000 “time” steps were calculated for each set. The oscillation behavior for the calcium and contractility concepts were chosen as selection criteria. If the amplitude of a concept in the step interval from 10,000 to 20,000 was less than 0.1/K, it was not considered as an oscillation. If, within the same interval, the amplitude value decreased by more then 10%, it was considered as a damped oscillation and the weight set was disregarded. The simulation had to demonstrate at least two full periods of oscillation between 10,000 and 20,000 “time” steps to be considered as successful.

#### Algorithm for testing hypotheses

The algorithm for evaluating the fitness index and ranking hypotheses consists of the following steps:

1. Define the phenotype as a set of experimental observations that the CMAP configuration should reproduce. These observations form the criteria against which the CMAP configurations are tested.
2. Build candidate CMAP configurations. Start with the elements that are known to be involved in the processes under study. Next, use all available knowledge to place connections (influences) between these elements.
3. Specify the weights that will be varied.
4. For each set of parameter weights, run a simulation with K = 5. Count the number of parameter combinations, *P_i_*, for each configuration that meets the criteria defined in step 1.
5. The value of *P_i_* is then used to calculate the fitness index of the configuration.
6. Hypotheses with the highest fitness indices are selected for further studies.
7. Control:
  - Start over for set sizes K = 3 and 7.
  - Perform Monte-Carlo simulations (see section ‘Monte-Carlo method for sampling parameter space’ in text) in which all parameter values are allowed to change.

The controls are needed to make sure that the results are not set size dependent or reflect a special choice of values for the fixed parameters.
